# Bioactivity and Antibacterial Analysis of Plasticized PLA Electrospun Fibers Reinforced with MgO and Mg(OH)_2_ Nanoparticles

**DOI:** 10.3390/polym16121727

**Published:** 2024-06-18

**Authors:** Adrián Leonés, Valentina Salaris, Laura Peponi, Marcela Lieblich, Alexandra Muñoz-Bonilla, Marta Fernández-García, Daniel López

**Affiliations:** 1 Instituto de Ciencia y Tecnología de Polímeros (ICTP-CSIC), C/Juan de la Cierva 3, 28006 Madrid, Spain; aleones@ictp.csic.es (A.L.); v.salaris@ictp.csic.es (V.S.);; 2 Centro Nacional de Investigaciones Metalúrgicas (CENIM-CSIC), 28040 Madrid, Spain

**Keywords:** electrospinning, polylactic acid, antibacterial properties, bioactivity, MgO NPs, Mg(OH)_2_ NPs

## Abstract

In this work, we focused on the bioactivity and antibacterial behavior of PLA-based electrospun fibers, efibers, reinforced with both MgO and Mg(OH)_2_ nanoparticles, NPs. The evolution of PLA-based efibers was followed in terms of morphology, FTIR, XRD, and visual appearance. The bioactivity was discussed in terms of hydroxyapatite growth after 28 days, considered as T28, of immersion in simulated body fluid, SBF. In particular, the biomineralization process evidenced after immersion in SBF started at T14 in both systems. The number of precipitated crystals increased by increasing the amount of both NPs. The chemical composition of the precipitated crystals was also characterized in terms of the Ca/P molar ratio after T28 of immersion in SBF, indicating the presence of hydroxyapatite on the surface of both reinforced efibers. Moreover, a reduction in the average diameter of the PLA-based efibers was observed, reaching a maximum reduction of 46 and 60% in the average diameter of neat PLA and PLA:OLA efibers, respectively, after 28 days of immersion in SBF. The antibacterial behavior of the MgO and Mg(OH)_2_ NPs in the PLA-based electrospun fibers was tested against *Escherichia coli*, *E. coli*, as the Gram-negative bacteria, and *Staphylococcus aureus*, *S. aureus*, as the Gram-positive bacteria, obtaining the best antibacterial activity against the Gram-negative bacteria *E. coli* of 21 ± 2% and 34 ± 6% for the highest concentration of MgO and Mg(OH)_2_ NPs, respectively.

## 1. Introduction

For the regeneration of tissues, biomaterials are required to treat damaged body areas. Ideally, biomaterials for tissue regeneration should display three main properties. The first is the ability to degrade under physiological conditions while remaining safe for human bodies [1]. The second is to resemble the mechanical properties associated with human tissue in terms of elastic modulus, tensile strength, and elongation at break [2]. Finally, biomaterials should display bioactivity, i.e., the ability to properly adapt to the biological environment, designed to stimulate appropriate cellular and tissue responses [3]. This could be achieved by the development of materials based on a polymeric nanocomposite [4]. In particular, combinations of biodegradable polymers and functional nanoparticles, NPs, offer numerous opportunities for improving mechanical properties and bioactivity.

Nanocomposite biomaterials can be processed by numerous techniques, such as solvent casting [5], 3D printing [6], or electrospinning [7]. In particular, the electrospinning technique obtains electrospun fibers, efibers, with a high surface area ratio, which properly recreates the extracellular matrix, enhancing the bioactivity of biomaterials. Among the biodegradable polymers used in electrospinning, poly(lactic acid), PLA, has been widely investigated for tissue engineering due to its biodegradability into non-toxic products under physiological conditions [8]. Additionally, the mechanical properties of PLA efibers can be enhanced by the addition of both organic and inorganic NPs, improving their elastic modulus, tensile strength, or elongation at break [9,10]. However, among the inorganic NPs, few studies have investigated the use of Mg-based NPs to enhance the bioactivity of electrospun biomaterials [1].

Magnesium, Mg, is a biocompatible metal that is already present in the human body in a concentration of 0.4 g of Mg·kg^−1^ [11]. Beyond their cooperative role with hydroxyapatite, HA, in maintaining bone health [12], Mg ions play an important role in mediating the functions of all cells in the body. In particular, Mg ions participate in cell functions such as attachment, proliferation, and migration [11,12]. Thus, the use of Mg in tissue engineering could potentially improve the bioactivity of nanocomposite biomaterials.

Among the Mg-based NPs available, magnesium oxide, MgO, and magnesium hydroxide, Mg(OH)_2_, NPs were used in our work because of their biocompatibility [13,14]. Furthermore, both MgO and Mg(OH)_2_ NPs show antimicrobial activity against bacteria, which can potentially reduce infections [15,16]. Many inorganic oxide NPs such as zinc oxide, ZnO, or titanium oxide, TiO_2_, NPs have shown antimicrobial properties against a broad spectrum of microorganisms [17]. However, these NPs cause significant concerns considering their toxicity due to the risks associated with heavy metal elements and their accumulation in the body [18]. 

In contrast, MgO and Mg(OH)_2_ NPs are considered an alternative to heavy metal oxide NPs because they can be efficiently degraded and metabolized inside the body, with Mg^2+^ and OH^-^ ions as final degradation products [13]. In addition, MgO and Mg(OH)_2_ NPs can enhance the mechanical properties of polymer efibers in terms of elastic modulus, tensile strength, or elongation at break [19]. Thus, some studies have recently been conducted investigating the addition of Mg-based NPs into polymer efibers for use in different fields such as environmental applications [20], energetic devices [21], or biomedical applications [22]. However, very few works involve the use of both MgO and Mg(OH)_2_ NPs in PLA efibers. In particular, on the one hand, Canales et al. [23]. added MgO nanoparticles at 10 and 20 wt%, comparing them with bioglass nanoparticles and a mix of both NPs, resulting in a 10 and 20 wt% of their final concentration with respect to the neat PLA matrix. They focused their attention on bone tissue regeneration, also studying cell viability for biomedical applications. They did not use plasticizer, and they did not take into account Mg(OH)_2_ nanoparticles. On the other hand, Salaris et al. [24] reported a comparative study of both NPs but in another matrix, such as Poly(ɛ-caprolactone) PCL, electrospun-fiber mats, observing the presence of monocalcium phosphate, dicalcium phosphate, and tricalcium phosphate, obtaining very low Ca/P values.

In this work, we focused on the bioactivity and antibacterial behavior of PLA-based efibers with both MgO and Mg(OH)_2_ NPs, comparing the results. The bioactivity will be discussed in terms of HA growth after 28 days of immersion in simulated body fluid, SBF, by analyzing the Ca/P ratio, SEM, and XRD results [25]. The antibacterial behavior of MgO and Mg(OH)_2_ NPs in PLA-based efibers was tested against *Escherichia coli*, *E. coli*, as Gram-negative bacteria, and *Staphylococcus aureus*, *S. aureus*, as Gram-positive bacteria, considering potential biomedical applications.

## 2. Materials and Methods

Poly(lactic acid) (PLA3051D, 3% of D-lactic acid monomer, molecular weight of 14.2 × 10^4^ g∙mol^−1^, density of 1.24 g∙cm^−3^) was supplied by NatureWorks^®^, Minneapolis, MN, USA. Lactic acid oligomer (Glyplast OLA8, ester content > 99%, density of 1.11 g∙cm^−3^, viscosity of 22.5 mPa∙s, molecular weight of 1100 g∙mol^−1^) was kindly supplied by Condensia Quimica SA, Barcelona, Spain. Magnesium oxide nanoparticles (MgO NPs, average particle size of 20 nm, 99.9% purity, molecular weight of 40.30 g∙mol^−1^) and magnesium hydroxide nanoparticles (Mg(OH)_2_ NPs, average particle size of 10 nm, 99.9% purity, density of 2.34 g·cm^−3^, molecular weight of 58.32 g∙mol^−1^) were supplied by Nanoshel LLC, Willmington, IL, USA.

Each solution was prepared following the process described in our previous work [26]. In particular, PLA pellets were previously dried in an oven overnight at 60 °C. The polymer solutions were used at a final concentration of 10 wt% in CHCl_3_:DMF (4:1). Both NPs were added at different concentrations with respect to the PLA matrix, such as 0.5, 1, 5, 10, 15, and 20 wt%. The different amounts of NPs were dispersed using a tip sonicator (Sonic Vibra-Cell VCX 750, Sonics & Materials, Newton, CT, USA), operating at 750 watts and an amplitude of 20% for 3 h. Once the different solutions were obtained, electrospun fiber mats were prepared in an Electrospinner Y-flow 2.2.D-XXX (Nanotechnology Solutions), following our previously described method [27].

The bioactivity of PLA-based electrospun fiber mats was studied by immersing a square of 1 cm^2^ of each PLA-based electrospun fiber system in simulated body fluid (SBF) for 28 days at 37 ± 1 °C to study the bioactivity at different immersion times, named T0, indicating the sample prior to immersion; T14, indicating the samples after 14 days of immersion; and T28, indicating the samples after 28 days of immersion in SBF. The SBF solution was prepared by following the protocol described by Kokubo et al. [25]. The extraction days are named T_x_, where x indicates the number of the corresponding day. The as-obtained electrospun mats are considered as time 0, T0, and are used as references. The samples were carefully washed with water and dried under vacuum for two weeks before characterization.

The antibacterial behavior of PLA-based electrospun fibers was studied against American Type Culture Collection (ATCC): *Escherichia coli* (*E. coli*, ATCC 25922), as Gram-negative bacteria, and *Staphylococcus aureus* (*S. aureus*, ATCC 29213), as Gram-positive bacteria, obtained from Oxoid^TM^. Microorganisms were incubated for 24 h at 37 °C. The optical density of the microorganism suspensions was measured in McFarland units, proportional to microorganism concentration, using a DEN-1B densitometer (Biosan, Madrid, Spain). The bacteria suspensions of about 10^8^ colony-forming units (CFU) were prepared by adjusting the concentration with saline solution to a McFarland turbidity standard of ca. 0.5. A suspension of ca. 5 × 10^3^ CFU∙mL^−1^ for each bacteria was finally obtained by further dilution with PBS. The antibacterial behavior of PLA-based electrospun fibers was determined following the E2149-20 standard method of the American Society for Testing and Materials [28]. Each sample was placed in a sterile falcon tube, and then the bacterial suspension was added. Falcon tubes containing only the inoculum were prepared as control experiments. The samples were shaken at 37 °C at 150 rpm for 24 h. Bacterial concentrations at time 0 and after 24 h were calculated using the counting method. Each sample was measured and counted at least twice.

Scanning electron microscopy, SEM, (PHILIPS XL30 Scanning Electron Microscope, Phillips, Eindhoven, The Netherlands) was used to study the morphology and the diameters of the efibers. The images are shown at ×8000 magnification, with a scale of 2 μm, 25.0 kV, and a spot size 3.0. All the samples were previously gold-coated (~5 nm thickness) in a Polaron SC7640 Auto/Manual Sputter Coater (Polaron, Newhaven, East Sussex, UK). The diameters were calculated as the average value of 30 random measurements for each sample using ImageJ 1.51k software. Energy-dispersive X-ray spectroscopy, EDX, analyses were carried out in a FE-SEM Hitachi SU 8000 (Hitachi, Tokyo, Japan) device with a Bruker XFlash Detector 5030 operating at 15 Kv.

Attenuated total reflectance-Fourier transform infrared spectroscopy (ATR-FTIR) measurements were conducted using a Spectrum One FTIR spectrometer (Perkin Elmer instruments, Shelton CT, USA). Spectra were obtained in the 4000–400 cm^−1^ region at room temperature in transmission mode, with a resolution of 4 cm^−1^ and an accumulation of 16 scans.

XRD measurements were performed using a Bruker D8 Advance instrument (Bruker, Billerica, MA, USA) with a CuK as the source (0.154 nm) and a Detector Vantec1 detector. The scanning range was 5° and 60°, and the step size and count time per step were 0.023851° and 0.5 s, respectively.

The results were statistically analyzed by one-way analysis of variance (ANOVA) and Tukey’s test, with a 95% confidence level, using the statistical computer package Statgraphics Centurion XVII v16.01 (Statpoint Technologies, Inc., Warrenton, VA, USA) [9].

## 3. Results

**Figure 1:**
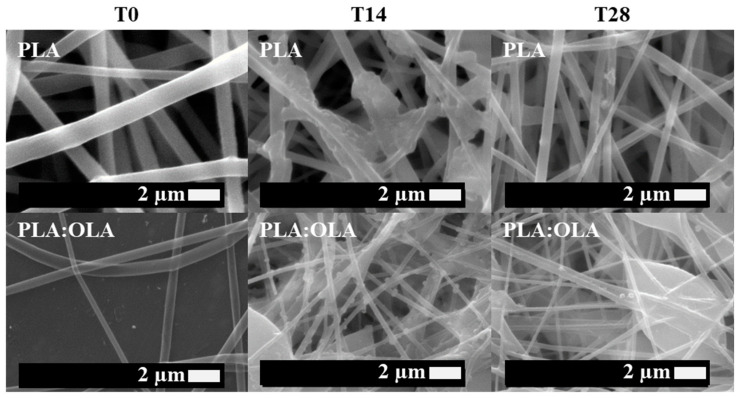
SEM images of PLA and PLA:OLA efibers in SBF at T0, T14, and T28.

**Figure 2:**
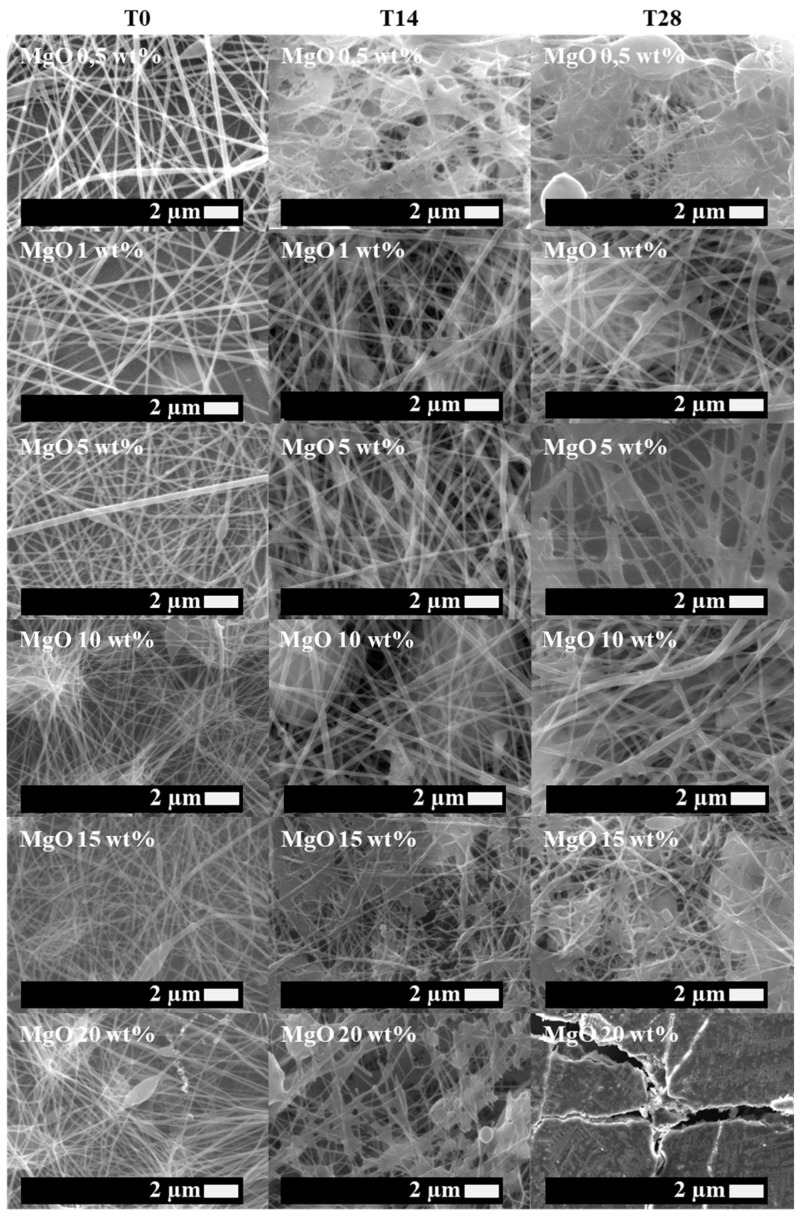
SEM images of PLA:OLA-MgO efibers in SBF at T0, T14, and T28.

**Figure 3:**
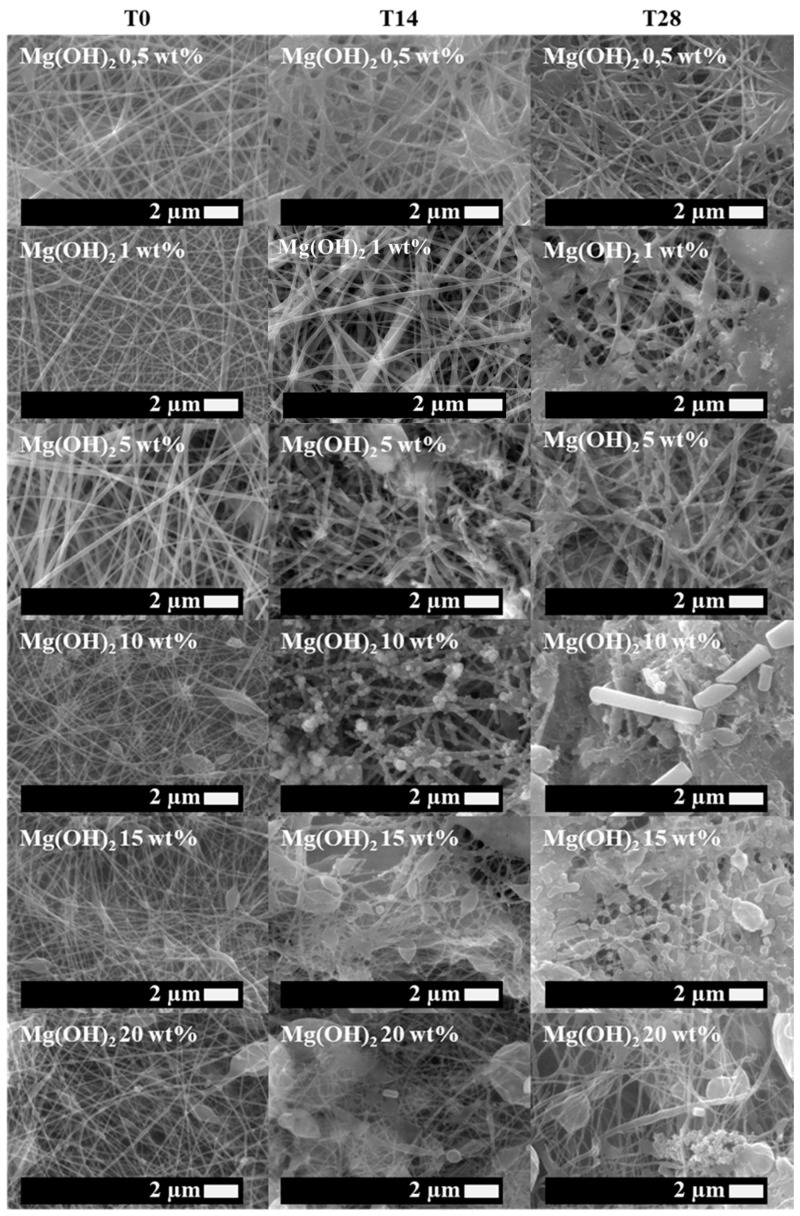
SEM images of PLA:OLA-Mg(OH)_2_ efibers in SBF at T0, T14 and T28.

First of all, neat PLA electrospun fiber mats, as well as plasticized PLA:OLA electrospun fiber mats, were successfully obtained. Moreover, different concentrations of MgO and Mg(OH)_2_ NPs, such as 0,5, 1, 5, 10, 15, and 20 wt%, were added to the polymeric solution, obtaining reinforced electrospun systems based on plasticized PLA. In this work, we focus on their bioactivity and antimicrobial response, comparing both systems. Therefore, in order to study the bioactivity of PLA-based efibers in terms of hydroxyapatite growth, SEM images of neat PLA, PLA:OLA, and PLA-based efibers with MgO and Mg(OH)_2_ NPs at T0, and after 14 and 28 days of immersion in SBF, are shown in Figure 1, Figure 2, and Figure 3, respectively.

As can be seen, the different efibers obtained by the electrospinning technique showed a 3D scaffold structure, with highly interconnected porosity. Both neat PLA and PLA:OLA efibers, our polymeric matrices at T0, showed smooth and regular surfaces, without the presence of beads. On the other hand, PLA:OLA-MgO and PLA:OLA-Mg(OH)_2_ efibers showed some beads as the amount of NPs increased from 5 wt%, due to the presence of NP agglomeration.

**Figure 4:**
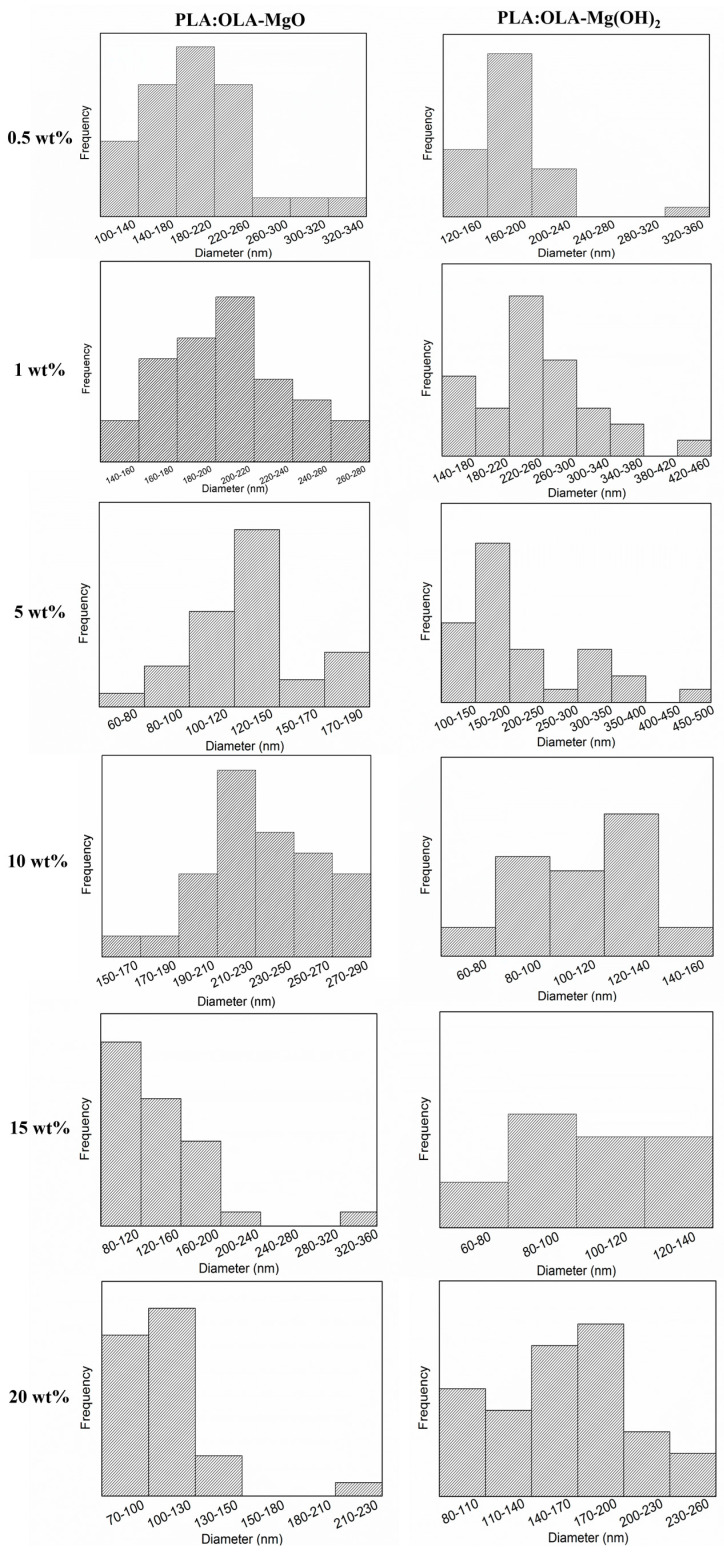
Diameter size distributions at T0 for the different electrospun fibers studied.

**Figure 5:**
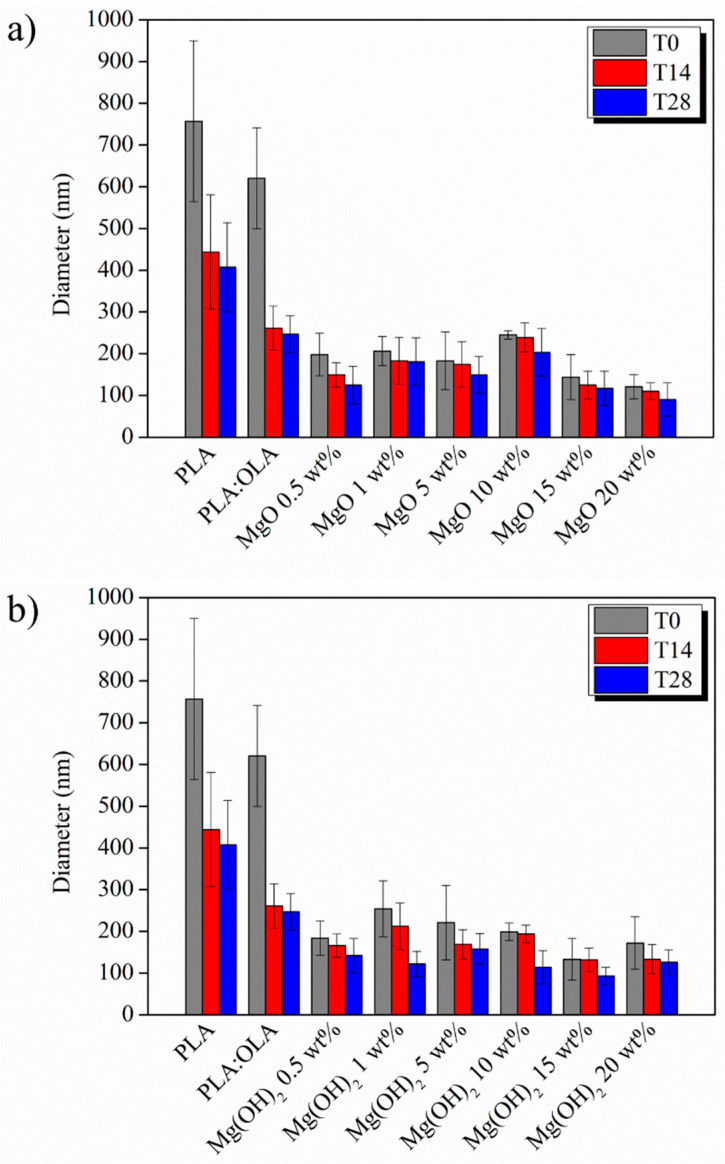
Average diameter evolution of PLA-based efibers in SBF at T0, T14, and T28 of (**a**) PLA:OLA-MgO and (**b**) PLA:OLA-Mg(OH)_2_ efibers.

In order to better visualize the fiber size distribution, in Figure 4, the diameter distribution of PLA and PLA:OLA efibers, as well as the different MgO and Mg(OH)_2_ electrospun nanocomposites at T0, is reported, while the evolution of the average diameter at the different immersion times is reported in Figure 5.

The biomineralization process, indicated by the presence of precipitated crystals on the surface of PLA-based efibers, started after 14 days of immersion in SBF in all PLA-based efibers, and continued until 28 days. In addition, it is worth noting that the immersion in SBF media affected the surface and morphologies of PLA-based efibers. Moreover, the amount of precipitated crystals increased by increasing the amount of both NPs, as can be observed in Figure 2 and Figure 3. In particular, for the PLA:OLA-MgO efibers, the presence of precipitated crystals started after 14 days of immersion in SBF, being more abundant after 28 days for PLA:OLA-MgO efibers, with NPs in the range of 5–20 wt%. On the other hand, in the PLA:OLA-Mg(OH)_2_ efibers, the precipitated crystals are clearly observed after 28 days in all the ranges of NP concentrations studied. The addition of both types of NPs, as well as the biomineralization process after immersion in SBF, affect the diameter evolution of PLA-based efibers, as can be observed in Figure 5 and as summarized in Table 1.

Simultaneously with the biomineralization process, a reduction in the average diameter of PLA-based efibers was observed, reported in Figure 5 and summarized in Table 1 in term of percentage of average diameter reduction (%D Reduction). In particular, after 28 days of immersion in SBF, a reduction of 46 and 60% in the average diameter of neat PLA and PLA:OLA efibers, respectively, was calculated. For PLA:OLA-MgO 0.5, 1, 5, 10, 15, and 20 wt% efibers, this reduction was noted as 37, 12, 19, 17, 18 and 25%, and for PLA:OLA-Mg(OH)_2_ 0.5, 1, 5, 10, 15, and 20 wt% efibers, it was 23, 52, 29, 42, 30, and 26%, respectively. This average diameter reduction indicated the simultaneous degradation of efibers under SBF conditions. This degradation in aqueous media is widely reported in the literature, indicating the breaking of polymeric chains by hydrolytic degradation [8].

The precipitated crystals on the surface of PLA-based efibers were characterized by EDX analysis. The chemical composition was analyzed, and the Ca/P molar ratio was calculated after 28 days of immersion in SBF for all efibers, as summarized in Table 2. In addition, for a better comparison, the Ca/P ratios of HA from different natural sources and different calcium phosphates are summarized in Table 3 and Table 4, respectively.

The precipitated crystals on the surface of neat PLA and PLA:OLA showed a Ca/P ratio of 3.33 and 2.83, respectively, which is not consistent with any calcium phosphates summarized in Table 2 and Table 3. However, this Ca/P ratio is described in the literature as carbonate apatite, if the hydroxyapatite is not carbonate-containing hydroxyapatite but carbonate apatite (according to the definition, carbonate ions occupy more than half the phosphoric acid positions), its Ca/P ratio should be much higher than the Ca/P ratio of 1.67 for the hydroxyapatite [29].

On the contrary, the presence of HA was confirmed by EDX on both PLA:OLA-MgO and PLA:OLA-Mg(OH)_2_ efiber systems, showing Ca/P ratios in the range of 1.25–1.73 [30]. It is important to note that Ca/P ratios range from 1.20 for amorphous calcium phosphate to 1.67 for HA compound. In fact, compared to the synthetic HAs summarized in Table 2, natural HAs data, collected in Table 3, are non-stoichiometric, since they contain traces of elements such as Na^+^, Zn^2+^, Mg^2+^, K^+^, Si^2+^, Ba^2+^, F^−^, and CO_3_^2−^, which make them similar to the chemical composition of human bone [31]. In our systems, the Ca/P ratios of HA extracted from different mammalian sources, presented in Table 3, are in the same range as those calculated for the PLA:OLA-MgO and PLA:OLA-Mg(OH)_2_ efibers, suggesting the appearance of HA on the surface of the PLA-based nanocomposites.

**Table 3:** Main Ca/P ratios of different calcium phosphates.

Source	Ca/P Ratio	Ref.
β-Tricalcium phosphate	1.50	[30]
Amorphous calcium phosphate	1.20–2.20	[30]
Hydroxyapatite deficient in calcium	1.50–1.67	[30]
Hydroxyapatite	1.67	[30]

**Table 4:** Main Ca/P ratios of HA from different natural sources.

Source	Ca/P Ratio	Ref.
Bovine bone	2.23–1.95	[32]
Camelus bone	1.65	[33]
Turkey femur bone	1.66	[34]
Porcine bone	1.64	[35]
Fish bone	1.65–1.83	[36]
Cow, goat, and chicken bone	1.57–1.65	[37]

**Figure 6:**
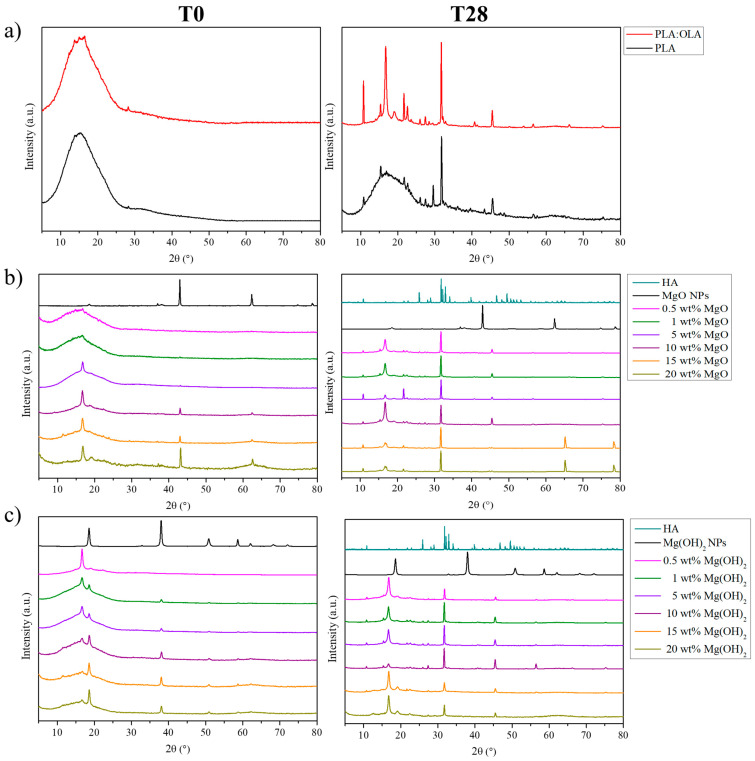
XRD patterns of (**a**) PLA and PLA:OLA, (**b**) PLA:OLA-MgO, and (**c**) PLA:OLA-Mg(OH)_2_ efibers in SBF at T0 (**left**) and T28 (**right**).

The precipitated crystals observed on the surface of the PLA-based efibers were characterized by XRD and FTIR analysis in order to determine their chemical structure. Specifically, XRD patterns of PLA-based efibers at T0 and T28 are reported in Figure 6.

At T0, previous to immersion in SBF, both neat PLA and PLA:OLA efibers show amorphous structures, whereas in the nanocomposites, the presence of the main peaks related to the crystallographic plane of MgO and Mg(OH)_2_ NPs can be clearly observed.

For PLA:OLA-MgO efibers, the MgO XRD pattern shows peaks at 2θ = 36.9°, 42.9°, 62.3°, 74.6°, and 78.6°, which are attributed to the [111], [200], [220], [311], and [222] crystallographic planes; all diffraction peaks can be indexed to the cubic crystalline system for MgO [38]. In particular, the peaks located at 42.9° and 62.3° increase by increasing the amount of MgO NPs in the efibers, and these were used to corroborate the presence of MgO NP in the efibers.

The Mg(OH)_2_ XRD pattern shows peaks at 2θ = 18.6°, 38.0°, 51.0°, and 58.6°, which are attributed to the [011], [101], [102], and [110] crystallographic planes and corresponded to the hexagonal structure of Mg(OH)_2_ [39]. Moreover, it is important to note the effect of both MgO and Mg(OH)_2_ NPs at 10 wt%. For MgO NPs, the crystallographic peak at 2θ = 18.7°, attributed to the [203] crystallographic plane of the α crystal of PLA [8], increased by increasing the amount of MgO NPs from 10 to 20 wt%, evidencing a higher crystallinity of the PLA:OLA-MgO efibers. However, this effect is not observed for Mg(OH)_2_ NPs; in this case, the crystallographic peak at 2θ = 18.7° decreased for the PLA:OLA-Mg(OH)_2_ efibers once it surpassed the 10 wt% of NPs.

The presence of peaks related to the crystallographic planes of the mineralization products is observed with both MgO and Mg(OH)_2_ NPs. To properly study those peaks related to HA, the main XRD peaks of PLA, MgO NPs, Mg(OH)_2_ NPs, and HA, as reported in the literature, are summarized in Table 5.

After 28 days of immersion in SBF, the PLA and PLA:OLA efibers showed crystallographic peaks at 2θ = 15.1, 16.6, 18.7, 22.1, 27.5, and 29.0°, which can be attributed to the [010], [200/110], [203], [015], [207], and [216] crystallographic planes of the α crystal conformation of PLA [8,40]. Comparing the XRD results before and after 28 days in SBF, we can conclude that immersion in SBF increases the degree of crystallinity in both the PLA and PLA:OLA efibers. An aqueous-based degradation media, such as SBF, provokes a hydrolytic reaction in the PLA chains, yielding a higher degree of crystallinity in PLA-based efibers [8]. This behavior was also observed in PLA:OLA-MgO and PLA:OLA-Mg(OH)_2_ efibers, and in addition, new crystallographic peaks appear after 28 days in SBF, which can be related to the presence of mineralization products. Firstly, it is important to note that the mineralization of carbonate apatite in the PLA and PLA:OLA samples is in good agreement with the Ca/P ratio previously described. The XRD patterns of HA and carbonated apatite are very similar; however, in carbonate apatite, the crystallographic peaks at 2θ = 26°, attributed to the [002] crystallographic plane of the hexagonal structure of HA, shifts to a lower value of 2θ = 25.5° [41]. In our PLA and PLA:OLA electrospun fibers, this crystallographic peak showed a higher intensity in comparison with those obtained for the electrospun fibers reinforced with both MgO and Mg(OH)_2_.

In particular, crystallographic peaks at 2θ = 22.0, 23.0, and 26.0° can be observed in both the PLA:OLA-MgO and PLA:OLA-Mg(OH)_2_ efibers, which are attributed to the [200], [111], and [002] crystallographic planes of the hexagonal structure of HA [42,43,44]. Moreover, a high peak at 2θ = 32.3° is observed, attributed to the presence of phosphate crystals (PO_4_^3−^) from HA [44]. Additionally, crystallographic peaks related to other mineralization products can be observed, such as 2θ = 45.0°, attributed to the [220] crystallographic plane of NaCl [45].

Salaris et al. [24] studied both MgO and Mg(OH)_2_ nanoparticles dispersed in PCL electrospun fiber mats. They observed the presence of monocalcium phosphate, dicalcium phosphate, and tricalcium phosphate, obtaining very low Ca/P values, varying from 0.18 at 0.5 wt% of MgO to 1.17 at 10 wt% of MgO and varying from 0.27 at 0.5 wt% of Mg(OH)_2_ to 0.43 at 10 wt% of Mg(OH)_2_. Only in the electrospun nanofiber mats reinforced with 20 wt% of NPs Ca/P obtained values of 1.76 and 1.33 for MgO and Mg(OH)_2_, respectively. This marks a significant difference from the results of our work, where we demonstrated that in plasticized PLA electrospun fiber mats, low concentrations of both MgO and Mg(OH)_2_ nanoparticles allow HA growth on the surface of the electrospun fiber mats. This fact is very important because it confirms that every system is different, and a deep study is required for each one in order to determine the correct response.

**Table 5:** Main XRD peaks of PLA, MgO NPs, Mg(OH)_2_ NPs, and HA reported in the literature.

	2θ (°)	Crystalline System	hkl	Ref. No.
	15.1	α crystals	010	[8,40]
	16.6	α crystals	200/110	[8,40]
	18.7	α crystals	203	[8,40]
	22.1	α crystals	015	[8,40]
	27.5	α crystals	207	[8,40]
	29.0	α crystals	216	[8,40]
	22.0	Hexagonal	200	[42,44]
	23.0	Hexagonal	111	[42,44]
	26.0	Hexagonal	002	[42,44]
	28.5	Hexagonal	102	[42,44]
	29.2	Hexagonal	210	[42,44]
	31.8	Hexagonal	211	[42,44]
	32.3	Hexagonal	112	[42,44]
	32.9	Hexagonal	300	[42,44]
	34.6	Hexagonal	202	[42,44]
MgO NPs	36.9	Cubic	111	[38]
	42.9	Cubic	200	[38]
	62.3	Cubic	220	[38]
	74.7	Cubic	311	[38]
	78.6	Cubic	222	[38]
Mg(OH)_2_ NPs	18.6	Hexagonal	001	[39]
	38.0	Hexagonal	101	[39]
	51.0	Hexagonal	102	[39]
	58.6	Hexagonal	110	[39]

**Figure 7:**
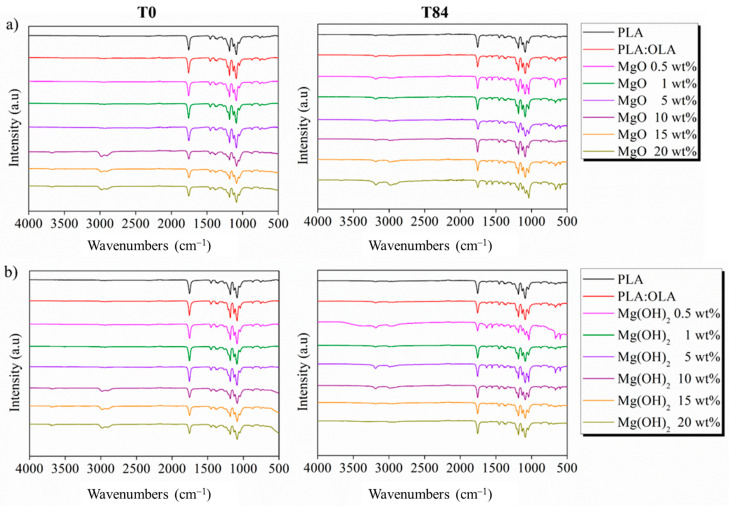
FTIR spectra of (**a**) PLA:OLA-MgO and (**b**) PLA:OLA-Mg(OH)_2_ efibers after immersion in SBF at T0 and T28. Spectra of PLA and PLA:OLA are also included in the graphs.

Once the XRD patterns of the PLA-based efibers were characterized, the functional groups and chemical interactions were studied by FTIR analysis, Table 6, and their spectra are shown in Figure 7. All PLA-based efibers showed the main characteristic FTIR bands of PLA at T0, in particular, the C=O stretching band at 1749 cm^−1^ attributed to the carbonyl group, the CH_3_ bond asymmetric vibration at 1452 cm^−1^, and the CH_3_ bond symmetric motion at 1182 cm^−1^, as well as both the C-O asymmetric stretching band and the C-O symmetric stretching band at 1128 cm^−1^ and 1084 cm^−1^, respectively [8,46]. Moreover, the presence of MgO and Mg(OH)_2_ NPs at high concentrations, i.e., 10, 15, and 20 wt%, increased the 2882–3000 cm^−1^ bands related to the C-H stretching (asymmetric, symmetric vibrations of –CH_3_ and CH modes) [47].

**Figure 8:**
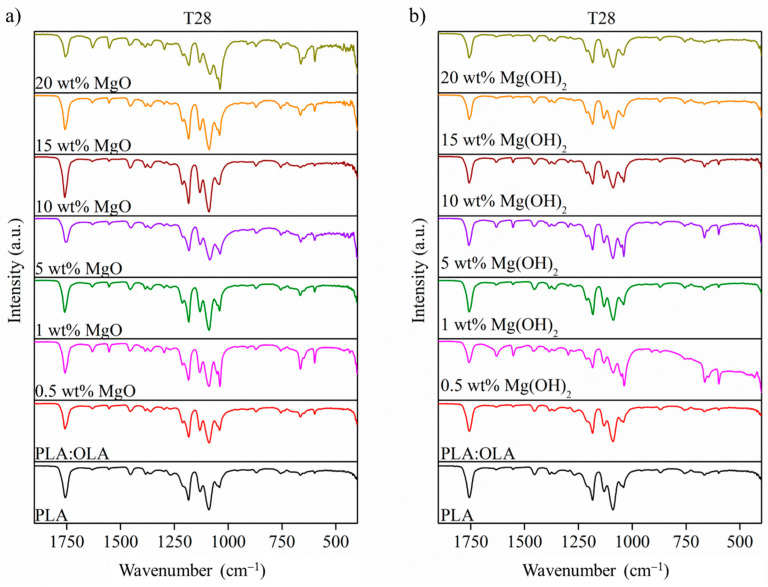
FTIR spectra 1800–400 cm^−1^ of (**a**) PLA:OLA-MgO and (**b**) PLA:OLA-Mg(OH)_2_ efibers after immersion in SBF at T28. Spectra of PLA and PLA:OLA are also included in the graphs.

After 28 days of immersion in SBF, a series of strong peaks can be seen in the spectra of both series, as reported in Figure 8, which could be related to HA, specifically, a new band at 575 cm^−1^ that can be associated with the antisymmetric bending of the PO_4_^3−^ groups [42,48]. In addition, the band at 650 cm^−1^ is well associated with the bending mode in the PO_4_^3−^ groups [42,48]. On the other hand, other mineralization products, such as carbonate, CO_3_^2−^, which showed a band at 1550 cm^−1^ associated with stretching mode in the CO_3_^2−^ groups [42,48], can be observed on the surface of the PLA-based efibers. These signals confirm the bioactivity of both the MgO and Mg(OH)_2_ efibers after immersion in SBF. Moreover, considering that SBF is an aqueous media, the hydrolytic degradation of PLA-based electrospun fibers after 28 days can be observed. Based in our previous results [8], the hydrolytic degradation through the PLA matrix provokes the breakage of the ester group, and new bands at 1650 cm^−1^, attributed to the carboxylate groups, can be observed for both the MgO and Mg(OH)_2_ NPs.

The antibacterial activity of the PLA-based electrospun nanocomposites was tested against *E. coli* (Gram-negative) and *S. aureus* (Gram-positive), and the relative antibacterial activity was calculated, as summarized in Table 7 and Table 8, respectively.

As expected, the results indicated that the neat PLA and PLA:OLA efibers did not show antibacterial activity against either *E. coli* or *S. aureus*, in agreement with previous results reported in the literature [49]. From the MgO and Mg(OH)_2_ NPs point of view, some considerations can be made. No statistically significant differences (*p* < 0.05) were observed in the relative antibacterial activity of fibers with MgO NPs at low concentrations such as 0.5, 1, 5, and 10 wt% against *E. coli* in comparison with neat PLA and PLA:OLA efibers. However, for the PLA:OLA-MgO 15 and 20 wt% efibers, a slight antibacterial activity percentage of 15 ± 2 and 21 ± 2 was obtained, respectively. The antibacterial activity of MgO NPs has been recently studied by some authors, with similar results. In particular, Swarrop et al. studied PLLA/MgO nanocomposite films, reporting 46% antibacterial activity against *E. coli* [50]. MgO NPs showed different antibacterial activity against *S. aureus* (Gram-positive). In general, no inhibitory effect was observed in the range of 0.5–5 wt%, but from this concentration on, the antibacterial activity starts to increase slightly. Thus, the PLA:OLA-MgO efibers were able to fight against Gram-negative bacteria more effectively than against Gram-positive bacteria.

**Figure 9:**
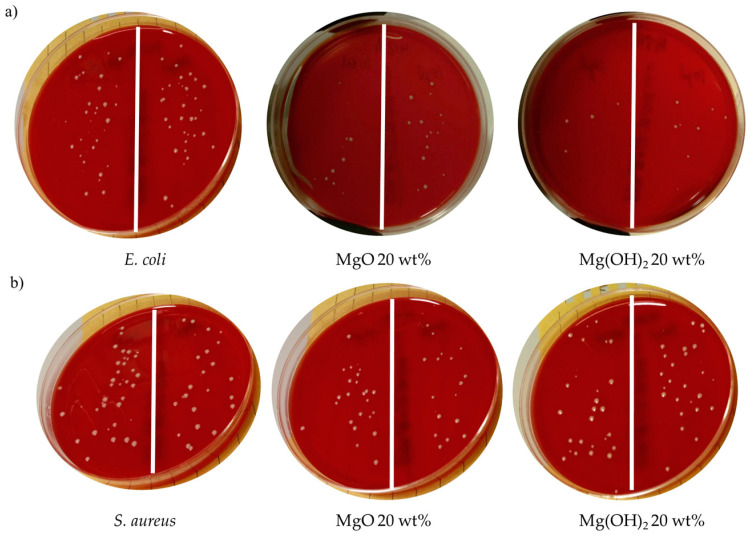
Images of the control plate and the plasticized PLA electrospun fiber at the highest concentrations of MgO and Mg(OH)_2_ NPs against (**a**) *E. coli* and (**b**) *S. aureus*.

On the other hand, Mg(OH)_2_ NPs showed statistically significant differences (*p* < 0.05) in the antibacterial activity against *E. coli* at concentrations in the range of 10–20 wt% in comparison with neat PLA and PLA:OLA. In particular, the achieved relative antibacterial activity of 16 ± 2, 17 ± 3, and 34 ± 6% for PLA:OLA-Mg(OH)_2_ 10, 15, and 20 wt% efibers, respectively, are slightly higher than those for PLA:OLA-MgO efibers in the same range of concentrations. In addition, poor antibacterial activity against *S. aureus* was observed for all PLA:OLA-Mg(OH)_2_ efibers. In Figure 9, qualitative comparison of the antibacterial activity obtained for PLA-based electrospun fibers at the highest concentration of both nanoparticles, that is 20 wt%, is reported, compared with the control for better visualization of the qualitative antibacterial activity of our PLA-based electrospun fibers. As far as we know, almost no investigations regarding the antibacterial activity of Mg(OH)_2_ NPs are found in the literature for comparison with our results. Only Meng et al. studied the antibacterial activity of Mg(OH)_2_ NPs against oral bacteria (*Streptococcus mutans*) and concluded that Mg(OH)_2_ NPs can be used to eradicate residual bacteria but with limited activity [15]. In our case, for a concentration at 20 wt% of both MgO and Mg(OH)_2_ NPs, the plasticized PLA-based electrospun nanocomposites show antibacterial activity. Regarding its use in potential biomedical applications, this result suggests an optimal range of NPs concentration of about 20 wt% for improving the antibacterial activity of PLA-based systems.

## 4. Conclusions

In this work, we studied the bioactivity and antibacterial behavior of PLA-based electrospun fibers, efibers, reinforced with both MgO and Mg(OH)_2_ nanoparticles, with different concentrations such as 0.5, 1, 5, 10, 15, and 20 wt%. The bioactivity was discussed in terms of hydroxyapatite growth at T28 of immersion in simulated body fluid, SBF. In particular, at T14, the biomineralization process is evidenced in both reinforced systems, at concentrations higher than 5 wt% for MgO NPs, and at all the concentrations studied for Mg(OH)_2_. However, the number of precipitated crystals increased by increasing the amount of both NPs. The chemical composition of the precipitated crystals was characterized in terms of Ca/P molar ratio after T28 of immersion in SBF, indicating the presence of hydroxyapatite on the surface of both reinforced efibers. Moreover, a reduction in the average diameter of PLA-based efibers was observed, reaching a maximum reduction of 46 and 60% in the average diameter of the neat PLA and PLA:OLA efibers, respectively, after 28 days of immersion in SBF, indicating their degradation process. The antibacterial behavior of MgO and Mg(OH)_2_ NPs in PLA-based electrospun fibers was tested against Escherichia coli, *E. coli*, as the Gram-negative bacteria, and *Staphylococcus aureus*, *S. aureus*, as the Gram-positive bacteria, obtaining the best antibacterial activity against the Gram-negative bacteria *E. coli* of 21 ± 2% and 34 ± 6% for the highest concentration of MgO and Mg(OH)_2_ NPs, respectively. This study suggests that these systems can potentially be used in biomedical applications.

## Figures and Tables

**Table 1:** Percentage of average diameter reduction (%D Reduction) for PLA, PLA-OLA, PLA:OLA-MgO, and PLA:OLA-Mg(OH)2 efibers after immersion in SBF.

Sample	%D Reduction	
	T14	T28
PLA	41	46
PLA:OLA	56	60
MgO 0.5 wt%	25	37
MgO 1 wt%	11	12
MgO 5 wt%	5	19
MgO 10 wt%	2	17
MgO 15 wt%	13	18
MgO 20 wt%	9	25
Mg(OH)_2_ 0.5 wt%	10	23
Mg(OH)_2_ 1 wt%	17	52
Mg(OH)_2_ 5 wt%	24	29
Mg(OH)_2_ 10 wt%	3	42
Mg(OH)_2_ 15 wt%	1	30
Mg(OH)_2_ 20 wt%	12	26

**Table 2:** Ca/P ratios of precipitated crystals in neat PLA, PLA-OLA, PLA:OLA-MgO, and PLA:OLA-Mg(OH)_2_ efibers.

Sample	Ca/P Ratio
	T28
PLA	3.33
PLA:OLA	2.83
MgO 0.5 wt%	1.5
MgO 1 wt%	1.66
MgO 5 wt%	1.73
MgO 10 wt%	1.4
MgO 15 wt%	1.5
MgO 20 wt%	1.33
Mg(OH)_2_ 0.5 wt%	1.26
Mg(OH)_2_ 5 wt%	1.25
Mg(OH)_2_ 10 wt%	1.6
Mg(OH)_2_ 15 wt%	1.5
Mg(OH)_2_ 20 wt%	1.5

**Table 6:** Most significant infrared bands related to HA and PLA.

	Frequency (cm^−1^)	Assignment	Ref. No.
Hydroxyapatite	567–604	P-O asymmetric vibrations of PO_4_^3−^ groups	[42,48]
	632 and 1625	Hydroxyl liberation mode	[42,48]
	418 and 575	Antisymmetric bending of PO_4_^3−^ groups	[42,48]
	1089–1039	Stretching mode in PO_4_^3−^ groups	[42,48]
	650	Bending mode in PO_4_^3−^ groups	[42,48]
	1550	Stretching mode in CO_3_^2−^ groups	[42,48]
PLA	2882 and 3000	Asymmetric–Symmetric vibrations of –CH_3_ and CH modes	[8,46]
	1452	CH_3_ bond asymmetric vibration	[8]
	1749	C=O stretching band	[8,46]
	1182	CH_3_ bond symmetric motion	[8,46]
	1128 and 1084	C-O asymmetric stretching band and C-O symmetric stretching band	[8,46]

**Table 7:** Relative antibacterial activity for PLA:OLA-MgO efibers.

Sample	*E. coli*	*S. aureus*
PLA	0 ^a^	0 ^a^
PLA:OLA	0 ^a^	0 ^a^
MgO 0.5 wt%	2 ± 2 ^a^	0 ^a^
MgO 1 wt%	2 ± 1 ^a^	0 ^a^
MgO 5 wt%	2 ± 2 ^a^	0 ^a^
MgO 10 wt%	5 ± 1 ^a^	6 ± 2 ^b^
MgO 15 wt%	15 ± 2 ^b^	5 ± 1 ^b^
MgO 20 wt%	21 ± 2 ^c^	5 ± 1 ^b^

Different letters in the column indicate significant differences, according to Tukey’s test (*p* < 0.05).

**Table 8:** Relative antibacterial activity for PLA:OLA-Mg(OH)_2_ efibers.

Sample	*E. coli*	*S. aureus*
PLA	0 ^a^	0 ^a^
PLA:OLA	0 ^a^	0 ^a^
Mg(OH)_2_ 0.5 wt%	1 ± 1 ^a^	0 ^a^
Mg(OH)_2_ 1 wt%	2 ± 1 ^a^	0 ^a^
Mg(OH)_2_ 5 wt%	2 ± 2 ^a^	0 ^a^
Mg(OH)_2_ 10 wt%	16 ± 2 ^b^	2 ± 1 ^b^
Mg(OH)_2_ 15 wt%	17 ± 3 ^b^	2 ± 1 ^b^
Mg(OH)_2_ 20 wt%	34 ± 6 ^c^	2 ± 1 ^b^

Different letters in the column indicate significant differences, according to Tukey’s test (*p* < 0.05).

## Data Availability

The raw data supporting the conclusions of this article will be made available by the authors on request.
